# Cohort Profile: The Zurich Primary HIV Infection Study

**DOI:** 10.3390/microorganisms12020302

**Published:** 2024-01-31

**Authors:** Matt C. Freind, Carmen Tallón de Lara, Roger D. Kouyos, David Wimmersberger, Hebert Kuster, Leonardo Aceto, Helen Kovari, Markus Flepp, Adrian Schibli, Benjamin Hampel, Christina Grube, Dominique L. Braun, Huldrych F. Günthard

**Affiliations:** 1 Department of Infectious Diseases and Hospital Epidemiology, University Hospital Zurich, 8091 Zurich, Switzerland; matt.freind@uzh.ch (M.C.F.); carmen.nathalia@gmail.com (C.T.d.L.); roger.kouyos@uzh.ch (R.D.K.); david.wimmersberger@usz.ch (D.W.); herbert.kuster@usz.ch (H.K.); dominique.braun@usz.ch (D.L.B.); 2 Institute of Medical Virology, University of Zurich, 8006 Zurich, Switzerland; 3 Center for Infectious Diseases, Klinik im Park, 8027 Zurich, Switzerland; leonardo.aceto@stadtspital.ch (L.A.); helen.kovari@hirslanden.ch (H.K.); markus.flepp@hin.ch (M.F.); 4 Department of Infectious Diseases, Hospital Epidemiology and Occupational Health, City Hospital Zurich, 8091 Zurich, Switzerland; adrian.schibli@stadtspital.ch; 5 Checkpoint Zurich, 8005 Zurich, Switzerland; benjamin.hampel@cpzh.ch (B.H.); christina.grube5@icloud.com (C.G.)

**Keywords:** HIV, primary HIV infection, acute HIV infection, recent HIV infection, acute retroviral syndrome, sexually transmitted diseases, cohort profile, clinical presentation

## Abstract

The Zurich Primary HIV Infection (ZPHI) study is a longitudinal cohort study established in 2002, aiming to study the clinical, epidemiological, and biological characteristics of primary HIV infection. The ZPHI enrolls individuals with documented primary HIV-1 infection. At the baseline and thereafter, the socio-demographic, clinical, and laboratory data are systematically collected, and regular blood sampling is performed for biobanking. By the end of December 2022, 486 people were enrolled, of which 353 were still undergoing active follow-up. Of the 486 participants, 86% had an acute infection, and 14% a recent HIV-1 infection. Men who have sex with men accounted for 74% of the study population. The median time from the estimated date of infection to diagnosis was 32 days. The median time from diagnosis to the initiation of antiretroviral therapy was 11 days, and this has consistently decreased over the last two decades. During the seroconversion phase, 447 (92%) patients reported having symptoms, of which only 73% of the patients were classified as having typical acute retroviral syndrome. The ZPHI study is a well-characterized cohort belonging to the most extensively studied primary HIV infection cohort. Its findings contribute to advancing our understanding of the early stages of HIV infection and pathogenesis, and it is paving the way to further improve HIV translational research and HIV medicine.

## 1. Introduction

The introduction and widespread use of antiretroviral treatment has drastically changed the course of the disease in people with HIV (PWH) [1]. With the intake of life-long combination antiretroviral treatment, PWH are now approaching a near-normal lifespan when compared to that of the general population [2]. Despite this progress, an effective vaccine or cure still seems distant, and successful treatment necessitates life-long therapy, regular monitoring, and is associated with long-term toxicity and costs.

Primary HIV infection (PHI), which represents the period of the first six months after the transmission event, plays a critical role in determining the course of the disease. The quick recognition of PHI presents an opportunity for an early diagnosis and prompt combination antiretroviral therapy (cART) initiation. cART not only interrupts the transmission chain, but helps also in preserving immune functions and minimizing the formation of a latent viral reservoir [3,4,5,6]. A better understanding of the processes that occur during PHI and the factors affecting its course could significantly advance the current understanding of the underlying biology that leads to HIV latency. This, in turn, could help in the development of effective therapies to eradicate the virus within the affected individuals. Studying the protective immune responses against HIV during PHI may also allow the development of effective vaccines in the future. It has been acknowledged that longitudinal studies of PWH shortly after PHI may be the key to a better understanding of HIV pathogenesis [6,7,8,9,10,11,12,13,14]. It is therefore imperative to not only identify PWH in this early stage, but also follow them longitudinally.

To enhance the current understanding of PHI and facilitate the aforementioned developments, the Zurich Primary HIV Infection Cohort Study (ZPHI) was established in 2002. We present the cohort profile for the 486 participants enrolled in this Zurich-based study since its start in 2002, with a focus on the socio-demographic, clinical, and laboratory characteristics, as well as the clinical manifestations of PHI.

## 2. Materials and Methods

### 2.1. Design

The ZPHI is an ongoing, open-label, longitudinal, non-randomized, and multi-site observational study (ZPHI, http://clinicaltrials.gov, 14 December 2023, NCT00537966). PWH are enrolled at the Department of Infectious Diseases and Hospital Epidemiology from the University Hospital, Zurich (USZ), Center for Infectious Diseases, Zurich, Checkpoint, Zurich, and the City Hospital, Zurich.

### 2.2. Primary Objectivies and Outcomes

The ZPHI focuses its research on PHI, focusing on its epidemiology, the effects of early cART on disease progression, researching the clinical presentations, as well as acting as a base for many clinical, translational, basic, and epidemiological research projects. The ZPHIs’ primary objectives are two-fold:

- To investigate and describe the factors which could influence the course of PHI and the factors that, in turn, could be influenced by PHI.
- To expand the established biobank to be used as a base for future research questions and projects.

### 2.3. Inclusion Criteria

The study prospectively recruits men and women aged 18 years and older who have been diagnosed with HIV-1 infection and have documented acute or recent infection, summarized as PHI.

Acute HIV-1 infection is defined as: a negative or evolving immunoblot result in the presence of positive p24 Ag and/or detectable plasma HIV-1 RNA, with documented HIV seroconversion within 90 days, with or without symptoms and/or clinical signs of PHI. Recent HIV-1 infection is defined as documented seroconversion within 180 days, but over 90 days; and/or an evolving immunoblot result after unambiguous transmission risk within 180 days; and/or a documented HIV infection and unambiguous transmission risk within 180 days; and/or a documented HIV infection and possible transmission risk within 180 days after infection and <0.5% ambiguous nucleotides [15]. All patients with a documented HIV-1 infection with an established diagnosis more than 180 days after the presumed date of infection are excluded from the study.

### 2.4. Data Collection and Project Measures

A standardized protocol is used for data collection at the ZPHI study institutions. At the baseline visit at the USZ, data are collected using an electronic case report form. The same paper form is utilized in the other study institutions. The data and biological samples are stored in a computer-based data management system and in a biobank, respectively.

At inclusion, a set of baseline data is collected. The ZPHI cohort baseline data cover socio-demographic, behavioral, clinical, laboratory, and viral data. Notably, the comprehensive detailed medical histories of PHIs are obtained, and an estimated date of infection (EDI) is determined. The EDI considers an array of assay reactivities (including the first positive and last negative HIV tests; negative, indeterminate, and positive Western blots; and positive p24 antigen and HIV-1 PCR), the PWHs’ reports of risk contacts, and the timing of the onset of acute retroviral syndrome (ARS) symptoms or other symptoms related to PHI. In addition to a complete medical history and physical examination, blood and urine samples are obtained for laboratory investigations, and the participants are screened for other sexually transmitted infections (STIs). Blood and, if available, stool specimens are additionally taken for the biobank.

The participants are followed up according to the study protocol, where clinical, therapeutic, and laboratory data are obtained. Systematic STI screening and interim viral loads and CD4/8 levels are also recorded. Blood is additionally collected for the biobank. In the first 48 weeks after inclusion, follow-up occurs frequently in line with the protocol. Subsequently, the collection of data and sample collection for the biobank are performed twice yearly until week 240 and once yearly thereafter.

All the patients in the ZPHI study, with the exception of the patients enrolled in the City Hospital, Zurich, are asked for a concurrent enrollment in the Swiss HIV Cohort Study (SHCS). The SHCS is a national, longitudinal, non-randomized, and multicenter observational study that has been ongoingly enrolling PWH since 1988 to monitor the HIV epidemic in Switzerland. By the end of 2019, 20,845 participants were registered, and 9816 participants were undergoing active follow-up. The SHCS covers at least 71% of all the patients on ART in Switzerland [16]. The SHCS study design has semi-annual visits, where sociodemographic, clinical, and laboratory information is collected. Certain data measures and biological samples are obtained within the frame of the SHCS and are thus not collected longitudinally in the ZPHI study for those patients concurrently enrolled in the SHCS cohort.

### 2.5. Ethical Considerations and Study Protocols

Participation in the study is voluntary, and the patients must provide written informed consent prior to inclusion. The study is conducted under the current version of the declaration of Helsinki, as well as complying to all national legal and regulatory requirements.

In 2002, the patients were initially enrolled in the Merck study 520 with a supplementary IC for the ZPHI study. Following two study extensions, the first version of the current protocol was submitted in 2007 and approved by the ethics committee of the canton of Zurich. In 2012, an amendment to the protocol was made to reflect the paradigm shift towards universal and life-long cART therapy [17,18,19,20]. Recently, a new full protocol of the ZPHI study was created, reflecting the changes in the management of HIV infection since the last ZPHI protocol amendment, and it was approved on 6 October 2023 by the ethics committee (ethical committee number 2023-01067). This protocol can be found in Appendix A. The duration of the recruitment phase was set to 25 years, with a reevaluation every 5 years, with the possible continuation of the study depending on the necessity for future research in the field of PHI.

### 2.6. Antiretroviral Treatment

Initially, all the ZPHI study participants with documented PHI were offered early cART with the standard first-line cART regimen of the time, regardless of the CD4 cell count or disease stage at initiation. The participants who did not wish to have early cART were enrolled in the observational study group. These groups enabled an investigation into the impact of early antiretroviral treatment on different disease parameters. After one year of suppressive early cART, the participants were given the option to stop cART, and thus, be switched to the observational study arm. This would allow the investigation of the impact of analytical treatment interruption (ATI) on various disease parameters. Since 2010, the treatment recommendations have changed towards a universal cART treatment for all the PWH, regardless of the CD4 cell count [17,18,19]. In light of this, a protocol amendment was made in 2012 to align with the shift towards universal life-long cART therapy, and all the PWH are recommended a life-long cART treatment. Between November 2015 and March 2017, 101 ZPHI participants were additionally enrolled into a pilot, randomized, controlled ART simplification trial, known as EARLY-SIMPLIFIED, consisting of patients who initiated cART during PHI and had <50 HIV-1 RNA copies/mL for at least 48 weeks [21,22]. The participants were randomized into a monotherapy group with dolutegravir 50 mg once daily and a control group receiving their current regimen consisting of an Integrase Strand Transfer Inhibitor (INSTI), a boosted protease inhibitor, or Non-Nucleoside Reverse Transcriptase Inhibitor (NNRTI), in combination with 2 Nucleoside Reverse Transcriptase Inhibitors (NRTIs).

## 3. Results

### 3.1. Enrolment, Rentention, and Mortality

**Figure 1:**
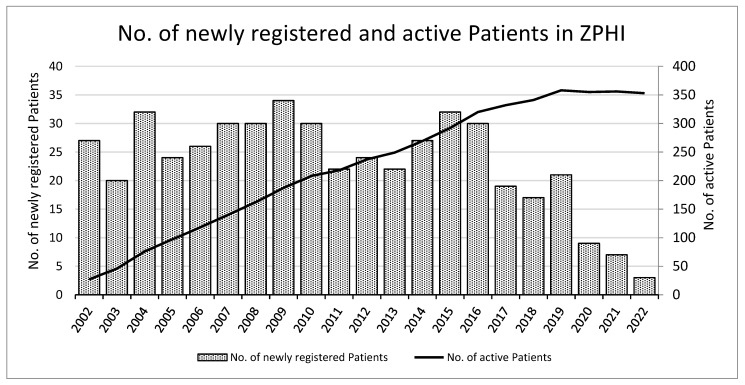
Number of newly registered and number of active patients in Zurich Primary Infection Study (ZPHI) seen in the respective calendar years.

Since 2002, the ZPHI had continuously enrolled PWH with documented acute or recent infection. By the end of 2022, a total of 486 participants had been registered at one of the ZPHI study institutions (Table 1). Of these 486 PWH, 478 (98%) were concomitantly enrolled in the SHCS. Four hundred and one (82.5%) out of the four hundred and eighty-six participants were enrolled at the University Hospital of Zurich. The number of newly enrolled PWH initially remained stable between 20 and 34 persons per year between 2002 and 2016, but since then, this has declined strongly, as illustrated in Figure 1. The yearly median number of PWH enrolled between 2002 and 2016 was 27.3, and this decreased to a median of 12.7 from 2017 to 2022.

Meanwhile, the number of active PWH in the ZPHI cohort reached a peak of 358 in 2019. As of end 2022, 353 were still undergoing active follow-up, while 133 were lost to the follow-up. Of those lost to the follow-up, 50 participants (37.6%) left the ZPHI network institutions, but remained in Switzerland, 44 participants (33.1%) emigrated, 19 participants (14.3%) did not respond to the invitation, 18 patients (13.5%) died, and 2 participants (1.5%) were lost for other reasons. Among the eighteen deaths, three were attributed to HIV infection, which included two cases of B cell lymphoma and one case of septic shock due to *Haemophilus influenzae* pneumonia. The remaining fifteen deaths were not directly associated with HIV and included four suicides and five deaths of unknown cause.

### 3.2. Socio-Demographic and Baseline Characteristics

The demographic and selected baseline characteristics of the ZPHI are presented in Table 1. Out of the 486 PWH ever registered, only 27 (5.6%) were women and 68 (14.0%) were non-white. The majority of patients (86.2%) had documented acute HIV-1 infection, while 13.8% had documented recent HIV-1 infection.

The self-reported presumed route of infection was reported as sexual contact between men who have sex with men (MSM) in 361 cases (74.3%) and heterosexual contact in 92 cases (18.9%). Intravenous drug use was identified as the presumed route of infection in twenty-five cases (5.1%), while accidental needle injuries were most likely responsible for three cases (0.6%).

**Figure 2:**
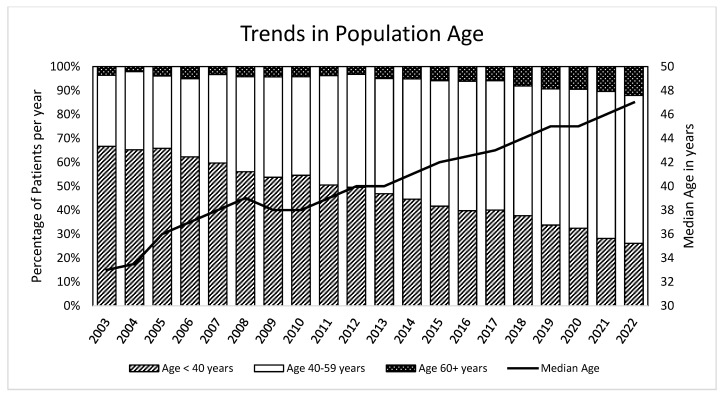
Age distribution in the in the Zurich Primary Infection Study (ZPHI) seen in the respective calendar years.

The median number of days from the estimated infection date to diagnosis was 32 days (IQR21-62). Broken down by PHI type, a median of 28 days was recorded for PWH with acute infection and 119 days for PWH with recent infection. The median age at diagnosis for all the participants was 34 years, and this has remained unchanged over time. However, PWH undergoing active follow-up have shown an increase in median age from 33 years in 2003 to 47 in 2022, as depicted in Figure 2.

**Figure 3:**
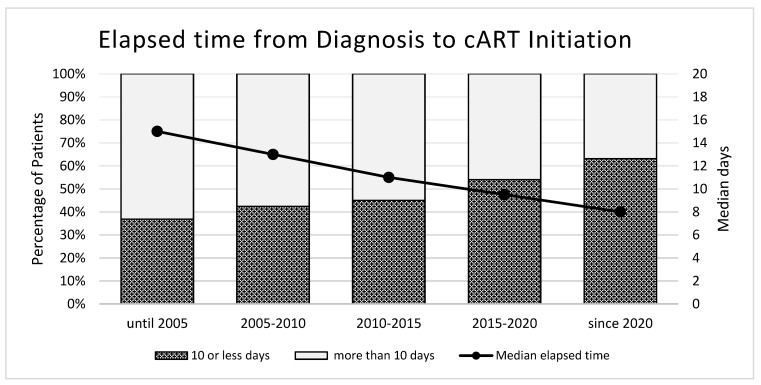
Time from HIV diagnosis to cART initiation over different time periods in the Zurich Primary Infection Study (ZPHI). cART, combination antiretroviral therapy.

The median time from diagnosis to the initiation of cART was 11 days, which has consistently decreased over the last two decades, as illustrated in Figure 3. All the participants, except for one who declined treatment, were exposed to cART, either as part of early ART, as part of the study protocol, or subsequent ART initiation according to the concurrent treatment guidelines at the respective times. As highlighted in Table 2, all except two active participants were on cART as of end 2022. One of these two untreated participants is an elite controller, who maintains an undetectable viral load without cART. A total of 97.5% of these active PWH had a viral load below 50 copies/mL as of end 2022. The median time to achieve a suppressed viral load of below 50 copies/mL after cART initiation was 89 days (IQR 56–138 days) for all the participants.

HIV-1 subtype B was detected in 328 cases (72.2%). Further analysis by route of infection revealed that 80.9% of the individuals with presumed MSM contact had HIV-1 subtype B, whereas only 46.5% of those who acquired HIV-1 by other means of transmission exhibited subtype B.

### 3.3. Presentation of PHI

The clinical and laboratory presentations of PHI are presented in Table 3. Out of the 486 participants, 92% presented with various symptoms of varying intensity and duration. A higher proportion of PWH with acute infections (92.8%) were symptomatic compared to those with recent infections (86.6%). A typical acute retroviral syndrome (ARS), as previously defined, was observed in 72.4% of the PWH [23]. Broken down, a typical ARS was more prevalent in those presenting with acute infection (73.9%) compared to those with recent infection (63.1%).

In all the participants, a fever was the most common symptom at PHI, with a prevalence of 72.6%, followed by malaise (65.3%) and pharyngitis (45.3%). The prevalence of each symptom was more frequent in the acute infection group, except for headaches and genital ulcers. Hospitalization was required for 15.8% of the PWH due to the severity of the symptoms.

The laboratory findings showed that 46.6% of the PWH had a lymphopenia, 27.8% had thrombopenia, and 24.8% had leukopenia. Elevated levels of AST and ALT were present in 21.3% and 31.4% of the PWH, respectively. The incidence of laboratory abnormalities, except for anemia, was higher in the PWH with acute infection than those with recent infection. A total of 25% of the participants presented with additional STIs at the baseline, with *Syphilis* (11.1%) and *Gonorrhea* (9.5%) being the most prevalent. Additionally, 12.1% presented with past hepatitis B or a chronic hepatitis B, as defined as positive hepatitis B surface antigens (HbsAg) and the absence of anti-Hbs antibodies (anti-HBsAb). HCV co-infection was present in 5.6%.

Opportunistic infections defined according to the Center for Disease Control and Prevention Classification were present in 28 (5.8%) patients at PHI.

The most common opportunistic infection was oropharyngeal candidiasis, which occurred in nineteen cases (3.9%), followed by six cases (1.2%) of esophageal candidiasis, three cases (0.6%) of immune-histochemistry-proven cytomegalovirus disease, and one case (0.2%) each of B cell lymphoma, cutaneous candidiasis, genital candidiasis, hairy leukoplakia, and pneumocystis jiroveci pneumonia.

### 3.4. Antiretroviral Treatment Simplification

In the EARLY-SIMPLIFIED pilot simplification trial, a total of 101 patients were enrolled, with 68 patients assigned to the dolutegravir monotherapy simplification group and 33 patients assigned to continue their combination antiretroviral therapy (cART) [21]. After 48 weeks, 67 patients in the dolutegravir monotherapy group achieved a virological response, compared to 32 out of 32 patients in the cART group. Of note, there was an absence of viral evolution, reflecting the emergence of drug resistance in the viral reservoir, as assessed in the proviral DNA from peripheral blood mononuclear cells [22]. This trend continued for 96 weeks, with 64 out of 64 patients maintaining a virological response in the dolutegravir monotherapy group and 30 out of 30 patients in the cART group [24]. Remarkably, at the study’s endpoint on week 192, there were no instances of virological failure reported in either group, demonstrating the non-inferiority of dolutegravir monotherapy as a simplification strategy [24].

## 4. Discussion

By the end of 2021, around 17,350 PWH were estimated to live in Switzerland [25]. The number of yearly new HIV infections in Switzerland has steadily decreased over recent years and reached a low of 283 in 2020 [25]. However, following the COVID pandemic, a mild increase in new infections was observed in 2021 because the frequency of HIV testing in the Swiss population was probably lower during the pandemic years, and there was a decreased number of instances of sexual contact [25,26]. The decline in new HIV infections in Switzerland in recent years despite the continuous decline of safer sex measures can be attributed to several factors, including the availability of pre-exposure prophylaxis PrEP, particularly as part of the SwissPrEPared-Program, reduced transmission due to universal HIV treatment, and enhanced identification due to accessible HIV testing [25,27]. The declining number of new infections highlights the success of HIV prevention and treatment efforts in Switzerland, but it also underscores the need to continue advocating for HIV awareness and testing. The decline in yearly HIV infections is reflected in the new enrollments in the ZPHI study, the total of which has been declining since 2015. The number of early new enrollments has decreased to fewer than 10 in the last three years, resulting in a slight decrease in active participants.

The HIV population in the ZPHI cohort is experiencing significant changes in their participant characteristics. One of the most prominent changes is the ageing population, as illustrated in Figure 2. As PWH age, it becomes increasingly important to consider factors, such as the long-term side effects of cART medications and the greater incidence and implications of comorbidities and comedication associated with ageing. The development of knowledge about how HIV and its therapies affect and interact with normal aging is still ongoing [28]. In light of the demographic shift towards aging among PWH, tackling these questions is crucial in order to further develop specific strategies that will allow PWH to age healthily.

Initially, the ZPHI study participants were offered early cART regardless of their CD4 cell count to investigate the impact of early antiretroviral treatment on different disease parameters. This early cART approach was initially controversial due to limited clinical data on its long-term effectiveness and potential side effects, the toxicity, treatment fatigue, as well as the cost and access of this treatment approach [8,10,29,30]. At the time, no guidelines recommended early cART initiation. After a year of early cART, the PWH were provided the choice to discontinue cART and transition into the observational group of the study. ATI was, however, discontinued since the effect of early cART on the set point viral load vanished after 3 years, and ongoing transmissions were observed after ATI [31,32]. Since 2010, the evidence and programmatic experience have increasingly favored the earlier initiation of cART, as it results in decreased morbidity, mortality, and reduced HIV transmission [17,18,19]. By 2015, also, the WHO guidelines reflected this paradigm shift with the treatment guidelines having shifted to universal cART for all PWH, irrespective of their CD4 count [19]. In line with this paradigm shift, increased efforts have been made towards rapid cART initiation. In the last 20 years, significant changes in the elapsed time between diagnosis and cART initiation in the ZPHI cohort have been observed, as demonstrated in Figure 3. Specifically, for the PWH diagnosed since 2005, the median time to cART initiation was 15 days, while the PWH diagnosed since 2020 had a median time of 8 days. A similar decrease has been observed in other cohorts around the world [33,34,35,36,37]. Whilst the ZPHI demonstrated a more rapid treatment start after diagnosis than many other similar cohorts in other countries, a few cohorts with programs and initiatives targeted to optimize cART initiation observed a median time from diagnosis to cART initiation as low as 2 days [33,34,35,36,37]. Early events between the virus and host and HIV pathogenesis during the early course of HIV infection are yet to be completely and definitely explained. However, very rapid cART initiation may have additional long-term implications in eliminating HIV within the host, reinforcing the importance to strive to continue to reduce the time from infection to cART start. Additionally, very rapid cART start could introduce opportunities to facilitate an HIV cure in the future if combined with the other novel interventions. A study conducted in Pattaya and Bangkok demonstrated that cART start within 5 days of HIV infection increased the likelihood of having undetectable HIV DNA in the central memory CD4 cells [38]. In summary, it has become clear over recent decades that HIV infection should be diagnosed as early as possible, and treatment should be initiated very rapidly. This is beneficial for the health of the individual, and also for the population because transmission is prevented (undetectable = untransmissible, U = U).

The EARLY-SIMPLIFIED study, which enrolled participants from the ZPHI cohort, stands out as one of the most significant contributions among the ZPHI endeavors. This study aimed to investigate the impact of monotherapy in individuals who initiated cART during the PHI phase. Its findings are particularly noteworthy, as they demonstrate the non-inferiority of once-daily dolutegravir compared to the standard cART [21]. Notably, there were no instances of virological failure observed during the study; this is a novel discovery that diverges from the prior trials, where dolutegravir monotherapy had been found inferior to cART. It is crucial to emphasize that these earlier studies involved patients who initiated cART during the chronic phase of HIV-1 infection, in contrast to the EARLY-SIMPLIFIED study, which focused on individuals who commenced cART during PHI [24]. This distinction suggests that future simplification studies should consider stratifying the participants based on the timing of HIV infection and timing of the initiation of their initial cART regimen. One possible explanation for the differing outcomes could be the approximately ten-fold lower latent HIV-1 reservoir observed in the patients who initiated cART early compared to those who initiated it during the chronic phase [22].. Identifying the patients who could benefit from monotherapy may lead to a reduction in antiretroviral drug-related toxicity and the associated costs. This paves the way for personalized medicine in HIV therapy, allowing for the stratification of patients’ treatment based on factors such as the time since infection. Such advancements underscore the vital role played by cohorts like ZPHI in advancing our knowledge and approach to HIV management.

A large proportion (27.6%) of the ZPHI participants presented with atypical ARS or were completely asymptomatic during the phase of seroconversion, which was more prevalent amongst the participants with recent infection (36.9%) than the participants with acute infection (26.1%). The atypical ARS presentation of certain individuals could result in difficulty in the recognition of PHI by healthcare professionals, which may result in a delayed diagnosis, and thus, later cART initiation. However, the PWH with atypical ARS often present as severely ill and are four times more likely to be hospitalized than the PWH with typical ARS [23]. The more severe presentation of the PWH with atypical ARS and increased hospitalization rate, may increase the likelihood of searching for an infectious cause, including HIV testing. The ZPHI study observed no delayed diagnosis in the participants with atypical ARS due to the aforementioned reasons [23]. Nonetheless, the recognition that a high proportion of PWH present atypically during PHI is vital to ensure that potential HIV infections are not missed and indicates the need for low-threshold HIV testing. This includes the testing in a hospital setting of severely ill patients with non-specific presentations, as well as non-symptomatic patients with reported risk factors.

At inclusion, a significant proportion of the participants (37.0%) exhibited co-infections, such as Chlamydia, Gonorrhea, hepatitis B, hepatitis C, Herpes simplex, and Syphilis. The recent observations have shown an alarming rise in the occurrence of STIs within the ZPHI population. Notably, MSM continue to be disproportionately affected by STIs, with 74.3% of the participants in the ZPHI presumed to have contracted infections through MSM contact. The challenge lies in the fact that many STIs remain asymptomatic, especially those affecting the pharynx and rectum [39,40,41]. Consequently, routine testing for STIs plays a critical role in identifying and treating a significant number of infections that would otherwise go undetected and untreated in asymptomatic individuals. Moreover, it is essential to address STIs concurrently with HIV, as they enhance the infectiousness of people living with HIV. Therefore, it becomes crucial to also combat the increasing prevalence of non-HIV STIs in order to effectively control the HIV pandemic.

The ZPHI study stands out as a well-characterized cohort and is among the largest and most extensively studied PHI cohorts in resource-rich settings. An extensive set of baseline data has been obtained, enabling the ZPHI to act as a base for a broad range of research topics. The main strength lies in its combination of clinical data and extensive longitudinal biobank. In addition to the pure clinical research [23,42,43], this approach facilitates investigations spanning a wide range of topics. These include studying the clinical and biological implications of co-infections [41,44,45,46], employing genetic and phylogenetic methods to examine the biological characteristics of transmitted viruses [47,48,49,50], conducting immunological studies to gain insights into HIV-specific immune responses [51,52,53,54], identifying the viral factors that may play a role in HIV-1 pathogenesis [49,55,56], as well as the description of the transmission of drug-resistant HIV-1 variants [49,55,57]. Additionally, the ZPHI is unique in that it assigns an estimated date of infection to each participant, making it ideal for research into early events between the virus and host and investigations into the long-term effects of early treatment and to study ART simplification studies such as the Early Simplified Study [21]. The 10 most important of all 71 so-far-published papers are summarized in Table 4 [15,23,24,32,41,49,55,58,59,60]. Additionally, the collaboration with the SHCS gives the ZPHI access to an abundance of data for research purposes. The ZPHI also has a standardized protocol, ensuring high-quality data are consistently collected.

The main weakness of the cohort is the limited number of participants, which makes the cohort not ideal for studying infrequent endpoints. The limited number of institutions involved in the ZPHI cohort means that the participants may sometimes be treated by non-ZPHI doctors, leading to dropouts. The cohort also has a low representation of females, heterosexual men, and ethnic groups, potentially limiting the generalizability of the results to these populations. Additionally, as a non-randomized observational study, the ZPHI is subject to inherent limitations, such as selection bias and unmeasured confounding, which should be considered when analyzing the data.

## Figures and Tables

**Table 1:** Demographic and selected baseline characteristics of Zurich Primary Infection Study (ZPHI).

ZPHI	Total	Acute Infection	Recent Infection
No. of patients ever registered	486 (100)	419 (86.2)	67 (13.8)
Sex ^a^			
Male patients	459 (94.4)	397 (94.8)	62 (92.5)
Female patients	27 (5.6)	22 (5.3)	5 (7.5)
Ethnicity			
White	418 (86.0)	365 (87.1)	53 (79.1)
Hispano-American	30 (6.2)	25 (6.0)	5 (7.4)
Black	21 (4.3)	18 (4.3)	3 (9.0)
Asian	9 (1.9)	5 (1.2)	4 (6.0)
Other/unknown	8 (1.6)	6 (1.4)	2 (3.0)
Patients under active follow-up			
Active patients	353 (72.6)	302 (72.1)	51 (76.1)
Loss to follow-up			
Total	133 (100)	117 (100)	16 (100)
Left ZPHI network	94 (70.7)	85 (72.6)	9 (56.3)
Patient did not respond to invitation	19 (14.3)	16 (13.7)	3 (18.8)
Death	18 (13.5)	15 (12.8)	3 (18.8)
Other	2 (1.5)	1 (0.9)	1 (6.3)
Center of registration			
University Hospital Zurich	401 (82.5)	347 (82.8)	54 (80.6)
Center for Infectious Diseases Zurich	47 (9.7)	35 (8.4)	12 (17.9)
Checkpoint Zurich	19 (3.9)	18 (4.3)	1 (1.5)
City Hospital Zurich	8 (1.6)	8 (1.9)	0 (0)
Arztpraxis Kalkbreite ^b^	7 (1.4)	7 (1.7)	0 (0)
Other	4 (0.8)	4 (1.0)	0 (0)
Most likely route of infection			
MSM	361 (74.3)	309 (73.7)	52 (77.6)
Heterosexual sex	92 (18.9)	80 (19.1)	12 (17.9)
Injection drug use	25 (5.1)	22 (5.3)	3 (4.4)
Needle injury	3 (0.6)	3 (0.7)	0 (0)
Other/unknown	5 (1.0)	5 (1.2)	0 (0)
Time from estimated Infection to diagnosis (days)			
Median	32	28	119
IQR	(21–62)	(20–45)	(101–142)
Age at HIV Diagnosis (years)			
Mean (median)	36 (34)	36 (35)	36 (33)
IQR	(28–42)	(28–42)	(26–43)
Opportunistic infections at diagnosis			
Total	28 (5.8)	23 (5.5)	5 (7.5)
Patients with CDC B-events	18 (3.7)	15 (3.6)	3 (4.5)
Patients with CDC C-events	10 (2.1)	8 (1.9)	2 (3.0)
HIV viral load at diagnosis (log copies/mL)			
Mean (median)	5.52 (5.49)	5.57 (5.58)	5.15 (5.08)
IQR	(4.71–6.53)	(4.80–6.59)	(4.51–5.89)
CD4^+^ cell count at diagnosis (cells/µL)			
Mean (median)	470 (409)	427 (408)	464 (418)
IQR	(297–533)	(290–521)	(337–582)
HIV Subtype			
B	328/454 (72.2)	284/391 (72.6)	44 (69.8)
CRF01_AE	40/454 (8.8)	35/391 (9.0)	5/63 (7.9)
A1	19/454 (4.2)	15/391 (3.8)	4/63 (6.3)
C	16/454 (3.5)	13/391 (3.3)	3/63 (4.8)
F1	13/454 (2.9)	12/391 (3.1)	1/63 (2.3)
Other	38/454 (8.4)	32/391 (8.2)	6/63 (9.5)
Time from estimated infection to treatment (days)			
Median	49	43	136
IQR	(31–90.5)	(29–71)	(123–179)
Time from HIV diagnosis to treatment (days)			
Median	11	11	18
IQR	(7–24)	(6–22)	(9–42)
Number of patients exposed to cART	485 (99.8)	418 (99.8)	67 (100)
CD4^+^ cell count at start of cART (cells/µL)			
Mean (median)	428 (389)	424 (381)	453 (406)
IQR	(288–532)	(286–525)	(310–547)
Time from cART initiation to suppressed viral load (under 50 copies/mL), (days)			
Mean (median)	103.2 (89)	108.4 (96.5)	67 (56)
IQR	(56–138)	(58–141)	(32–87)

MSM, Men who have sex with men; IQR, interquartile range; CDC, Centers for Disease Control and Prevention; B-event and C-event, HIV disease progression classified on the classification system of the CDC; cART, combination antiretroviral therapy. ^a^ Defined as sex at birth. No transgender or non-binary persons were enrolled, but if so, they could also be enrolled. ^b^ Prior to the new protocol, Arztpraxis Kalkbreite was also a study center.

**Table 2:** Characteristics of active participants of Zurich Primary Infection Study (ZPHI) seen in the respective calendar years.

ZPHI	2002	2007	2012	2017	2022
No. of active patients	27	140	238	332	353
Gender					
Male patients	22 (81.5)	124 (88.6)	224 (94.1)	313 (94.3)	336 (95.2)
Female patients	5 (18.5)	16 (11.4)	14 (5.9)	19 (5.7)	17 (4.8)
Ethnicity					
Caucasian	24 (88.9)	122 (87.1)	209 (87.8)	290 (87.3)	310 (87.8)
Hispano-American	0 (0)	8 (5.7)	13 (5.5)	18 (5.4)	16 (4.5)
Black	2 (7.4)	7 (5)	11 (4.6)	13 (3.9)	12 (3.4)
Asian	1 (3.7)	2 (1.4)	3 (1.3)	6 (1.8)	8 (2.3)
Other/unknown	0 (0)	1 (0.7)	2 (0.8)	5 (1.5)	7 (2.0)
Most likely route of infection					
MSM	13 (48.1)	100 (71.4)	187 (78.6)	256 (77.1)	270 (76.5)
Heterosexual sex	12 (44.4)	34 (24.3)	43 (18.1)	63 (19.0)	57 (16.2)
Injection drug use	0 (0)	3 (2.1)	4 (1.7)	8 (2.4)	19 (5.4)
Needle injury	2 (7.4)	3 (2.1)	3 (1.3)	2 (0.6)	2 (0.6)
Other/unknown	0 (0)	0 (0)	1 (0.4)	3 (0.9)	5 (1.4)
Age (years)					
Mean (median)	36.3 (33)	39.6 (39)	40.9 (40)	43.9 (44)	47.2 (47)
Infection type at diagnosis					
Acute infection	25 (92.6)	126 (90)	212 (89.1)	284 (85.5)	302 (85.6)
Recent infection	2 (7.4)	14 (10)	26 (10.9)	48 (14.5)	51 (14.4)
Viral load < 50 cps/mL (%)	16 (59.3)	88 (62.9)	208 (87.4)	325 (97.9)	344 (97.5)
Viral load < 400 cps/mL (%)	19 (70.4)	95 (67.9)	222 (93.3)	331 (99.7)	350 (99.2)
On cART	21 (77.8)	131 (93.6)	234 (98.3)	330 (99.4)	351 (99.4)

MSM, Men who have sex with men; cART, combination antiretroviral therapy.

**Table 3:** Clinical and baseline laboratory manifestations and presentations of PHI in the Zurich Primary Infection Study (ZPHI).

	All N = 486	Acute N = 419	Recent N = 67
General PHI Manifestation			
Symptomatic	447/486 (92.0)	389/419 (92.8)	58/67 (86.6)
Typical ARS	347/479 (72.4)	306/414 (73.9)	41/65 (63.1)
Atypical ARS	100/479 (20.9)	83/414 (20.0)	17/65 (26.2)
Hospitalisation	77/486 (15.8)	73/419 (17.4)	4/67 (6.0)
Clinical Manifestation			
Fever	352/485 (72.6)	318/419 (75.9)	34/66 (51.5)
Malaise	305/467 (65.3)	273/407 (67.1)	32/60 (53.3)
Rash	198/483 (41.0)	181/417 (43.4)	17/66 (25.8)
Headache	178/472 (37.7)	153/408 (37.5)	25/64 (39.1)
Cough	94/480 (19.6)	82/416 (19.7)	12/64 (18.8)
Lymphadenopathy	207/478 (43.3)	182/413 (44.1)	25/65 (38.5)
Pharyngitis	214/472 (45.3)	194/410 (47.3)	20/62 (32.3)
Myalgia	143/447 (32.0)	131/387 (33.9)	12/65 (18.5)
Arthralgia	89/446 (20.0)	79/386 (20.5)	10/65 (15.4)
Vomiting	61/472 (12.9)	57/407 (14.0)	4/65 (6.2)
Night sweats	156 /477 (32.7)	141/412 (34.2)	15/65 (23.1)
Weight loss	189 /468 (40.4)	170/403 (42.2)	19/65 (29.2)
Diarrhea	167/474 (35.2)	152/405 (37.2)	15 /65 (23.1)
Nausea	124/469 (26.4)	115/405 (28.4)	9/64 (14.1)
Neurological symptoms	52/485 (10.7)	48/419 (11.5)	4/66 (6.1)
Oral ulcers	62/470 (13.2)	54/404 (13.4)	8/66 (12.1)
Genital ulcers	27/462 (5.8)	20/397 (5.0)	7/65 (10.8)
Laboratory Findings			
Anemia	76/476 (16.0)	65/411 (15.8)	11/65 (16.9)
Thrombopenia	133/479 (27.8)	125/414 (30.2)	8/65 (12.3)
Neutropenia	42/450 (9.3)	41/389 (10.5)	1/61 (1.6)
Leukopenia	119/479 (24.8)	118/414 (28.5)	1/65 (1.5)
Lymphopenia	217/466 (46.6)	199/403 (49.4)	18/63 (28.6)
Elevated AST	97/455 (21.3)	86/393 (21.9)	11/62 (17.7)
Elevated ALT	148/472 (31.4)	132/409 (32.3)	16/63 (25.4)
Co-infections			
Chlamydia	29/454 (6.4)	23/396 (5.8)	6/60 (10)
Gonorrhea	44/462 (9.5)	38/404(9.4)	6/58 (10.3)
Hepatitis B	58/478 (12.1)	53/413 (12.8)	5/65 (7.7)
Hepatitis C	26/463 (5.6)	21/398 (5.1)	5/65 (7.7)
Herpes simplex virus I+II	11/458 (2.4)	8/400 (2)	3/58 (5.2)
Syphilis	53/476 (11.1)	40/412 (9.7)	13/64 (20.3)
Total Patients with co-infections	179/484 (37.0)	149/418 (35.6)	30/66 (45.5)

PHI, primary HIV infection; ARS, acute retroviral syndrome. Typical ARS and atypical ARS have previously been defined by Braun et al. [23].

**Table 4:** Top 10 papers of the Zurich Primary Infection Study (ZPHI).

Paper	Design	Methods	Main Outcome	Reference
Delay of HIV-1 rebound after cessation of antiretroviral therapy through passive transfer of human neutralizing antibodies	Phase I/II study	Infusion of three neutralizing antibodies (2G12, 2F5 and 4E10) for studying suppression of viral rebound upon stop of antiretroviral treatment	Antibody-treated individuals had delayed rebound to a control group of acutely infected patients without Ab	Trkola et al. Delay of HIV-1 rebound after cessation of antiretroviral therapy through passive transfer of human neutralizing antibodies. *Nature Medicine*. 2005:11:615-622 [58].
HIV-1 transmission after cessation of early antiretroviral therapy among men having sex with men	Molecular epidemiological population based study to identify transmissions originating from patients stopping ART	pol sequences from genotypic resistance tests and clonal env C2-V3-C3 sequences	Five new primary infection events originating from ZPHI patients within 16–61 weeks after stopping early ART	Rieder et al. HIV-1 transmission after cessation of early antiretroviral therapy among men having sex with men. *AIDS*. 2010;24:1177-1183 [32].
Ambiguous Nucleotide Calls From Population-based Sequencing of HIV-1 are a Marker for Viral Diversity and the Age of Infection	HIV sequence analysis of PWH and known infection date	Sequence analysis of partial pol sanger sequences from PWH and known infection date determining the fraction of ambiguous nucleotides	Fraction of ambiguous nucleotides is a useful marker for the age of infection	Kouyos et al. Ambiguous Nucleotide Calls From Population-based Sequencing of HIV-1 are a Marker for Viral Diversity and the Age of Infection. *Clinical Infectious Diseases*. 2011;52(4):532–539 [15].
Origin of minority drug-resistant HIV-1 variants in primary HIV-1 infection	Phylogenetic analysis	Allele specific polymerase chain reaction for drug resistance mutations in 204 acute or recent seroconverters and phylogenetic linkage to chronically infected PWH	Drug-resistant minority variants can be transmitted	Metzner et al. Origin of minority drug-resistant HIV-1 variants in primary HIV-1 infection. *The Journal of Infectious Diseases*. 2013;208(7):1102–1112 [55].
Frequency and Spectrum of Unexpected Clinical Manifestations of Primary HIV-1 Infection	Prospective collection of clinical signs and symptoms during HIV acute infection	Characterizing typical and atypical belonging to the acute retroviral syndrom’s symptoms and signs	Unexpected clinical presentations occurred in a large fraction of PWH and a primary HIV infection	Braun et al. Frequency and Spectrum of Unexpected Clinical Manifestations of Primary HIV-1 Infection. *Clin Infect Dis*. 2015 Sep 15;61(6):1013–1021 [23].
Tracing HIV-1 transmission: envelope traits of HIV-1 transmitter and recipient pairs	Comparison of phenotypic and genotypic viral characteristics of transmission pairs	Identification of probably transmission pairs, retrieving plasma and cellular samples from the biobank and phenotype and genotype HI-Virus of transmitter and recipients	No clear distinction of transmitter/founder virus from their matched transmitter viruses, suggesting stochastic transmission	Oberle et al. Tracing HIV-1 transmission: envelope traits of HIV-1 transmitter and recipient pairs. *Retrovirology*. 2016;13(1):62 [49].
The interplay between replication capacity of HIV-1 and surrogate markers of disease	Determination of replication capacity of 355 whole-genome primary HIV-1 isolates on primary cells	Correlation analysis between disease parameters and RC	RC correlated with set point viral load and showed significant differences between subtypes studied	Rindler et al. The interplay between replication capacity of HIV-1 and surrogate markers of disease. *J Infect Dis*. 2022; *226* (6), 1057–1068 [59].
Sustained viral suppression with dolutegravir monotherapy over 192 weeks in patients starting combination antiretroviral therapy during primary HIV infection (EARLY-SIMPLIFIED): a randomized, controlled, multi-site, non-inferiority trial	Randomized, open label, non-inferiority trial	2:1 randomization DTG monotherapy vs. continuation of cART among people starting fist cART during PHI with at least 48 weeks viral load suppression	Non-inferiority of DTG monotherapy vs. cART after 5 years of follow-up	West et al. Sustained viral suppression with dolutegravir monotherapy over 192 weeks in patients starting combination antiretroviral therapy during primary HIV infection (EARLY-SIMPLIFIED): a randomized, controlled, multi-site, non-inferiority trial. *Clinical Infectious Diseases*. 2023; *77*, 1012–1020 [24].
High Rates of Subsequent Asymptomatic Sexually Transmitted Infections and Risky Sexual Behaviour in Patients Initially Presenting With Primary Human Immunodeficiency Virus-1 Infection	Prospective longitudinal data collection for sexually transmitted infections (STI)’s	Systematic screening for STI’s with assessment of prevalence, incidence and clinical presentation and identification of risk factor	Very high point prevalence and incidence of STI’s with the vast majority being asymptomatic	Braun et al. High Rates of Subsequent Asymptomatic Sexually Transmitted Infections and Risky Sexual Behavior in Patients Initially Presenting With Primary Human Immunodeficiency Virus-1 Infection. *Clin Infect Dis*. 2018 25;217(12):1883–1888 [41].
Dolutegravir monotherapy as maintenance strategy: A meta-analysis of individual participant data from randomized controlled trials	Individual participant meta-analysis	Combining four randomized controlled trials evaluating dolutegravir monotherapy versus combined antiretroviral therapy (cART) among virologically individuals controlled for at least 6 months on cART	Significantly increased risk for viral failure on dolutegravir monotherapy compared to cART with D4 T cells nadir <350 mm^3^ having the highest adjusted hazard ratio for viral failure.	Fournier et al. Dolutegravir monotherapy as maintenance strategy: A meta-analysis of individual participant data from randomized controlled trials. *Open Forum Infectious Diseases*. 2022;9(6),ofac107 [60].

## Data Availability

Data are available upon request.

## Appendix A

The amended ZPHI protocol was approved on 6 October 2023 and has been included in the Appendix.
